# Prediction of neoadjuvant chemotherapy response using diffuse optical spectroscopy in breast cancer

**DOI:** 10.1007/s12094-017-1745-8

**Published:** 2017-09-18

**Authors:** Ying-hua Yu, Xiao Zhu, Qin-guo Mo, Ying Cui

**Affiliations:** 1 Department of Breast Surgery, The Affiliated Tumor Hospital of Guangxi Medical University, NO. 71, He Di Lu, Nanning, Guangxi 530021 People’s Republic of China; 2 The Graduate School, The Affiliated Tumor Hospital of Guangxi Medical University, NO. 71, He Di Lu, Nanning, Guangxi 530021 People’s Republic of China

**Keywords:** Near-infrared diffuse optical spectroscopy, Neoadjuvant chemotherapy response, Blood-oxygen content, Prediction, Accuracy

## Abstract

**Purpose:**

Near-infrared diffuse optical spectroscopy (DOS) has been recently used to predict neoadjuvant chemotherapy response (NAC). In the present study, we explore the change in blood-oxygen content using DOS to predict NAC response against breast cancer.

**Materials and methods:**

A total of 20 patients were enrolled and underwent DOS scan with blood-oxygen detection before each treatment cycle. The first DOS scan was performed before NAC treatment (pretreatment), and subsequent scans were performed after each NAC treatment circle. Changes in blood content and oxygen content by DOS were evaluated and compared with tumor size, and their changes were analyzed in response versus nonresponse group.

**Results:**

Thirteen patients were classified into response and seven patients into nonresponse group. The tumor blood content value (−1.06 ± 0.43) and oxygen content value (0.48 ± 0.17) of DOS at pretreatment was significantly different from presurgery in response group (*P* < 0.05), but not in nonresponse group. In response group, the percentage change in blood content (median 91.19%) was significantly larger than tumor size (median 48.89%) (*P* = 0.0035), while in oxygen content (median 47.11%) is not (*P* = 0.2815). Comparing each cycle, the percentage change in blood content could distinguish responder from non-responder as early as after the first treatment cycle (19.1 versus 6.6%, *P* = 0.0265). Blood content percentage sensitivity was 76.9% and specificity was 85.7% (AUC 0.912), while oxygen content percentage sensitivity was 76.9% and specificity was 71.4% (AUC 0.797).

**Conclusion:**

Both blood and oxygen content measured by DOS could be used to discriminate responder to the treatment versus non-responder. Among the two, percentage change of blood content was more precise and earlier than that of oxygen content to predicted breast tumor response. The percentage change in blood content could distinguish responder from non-responder after the first treatment cycle.

## Introduction

Breast cancer is considered one of the major cause of cancer-related mortality in women worldwide. Five percent of newly diagnosed breast cancers is locally advanced breast cancer (LABC) [8]. Women with LABC commonly have poor outcomes and required neoadjuvant chemotherapy (NAC) before surgery. As one important therapy of multimodality LABC treatment, NAC can either decrease large tumor bulk before mastectomy or allow breast-conserving therapy. Furthermore, after NAC, those patients with pathologic response have better prognosis than those without a response [2, 5]. Therefore, predicting the tumor response to NAC is critical in the multimodality LABC treatment and chemotherapy regimens could be changed in advance in case of a predicted poor response.

Nowadays, many methods are available to assess tumor response, such as clinical palpation, ultrasound [18], X-ray mammography, magnetic resonance imaging (MRI) [21], and positron emission tomography/computed tomography (PET/CT) [3]. However, they still have their limitations. Clinical palpation and ultrasound are not sensitive enough to detect tumor response. X-ray mammography is not indicated in presence of high mammographic density [12] or fibrotic tissue [15], and it has poor correlation with pathology [20]. Compared to other method, MRI and PET/CT have high sensitivity to monitor LABC treatment response. However, it lacks standard dynamic contrast-enhanced criteria and remains relatively costly.

Thus, an inexpensive and noninvasive diagnostic method is needed to monitor NAC response. Near-infrared diffuse optical spectroscopy (DOS) has been recently considered in breast cancer diagnosis. This imaging modality came from optical imaging [4] and did not gain clinical interest because of poor differentiation between tumor and benign tissue. With advances in detection technology and computing, DOS could measure near-infrared absorption and scattering spectra across tissue. This method can reflect the change of deoxyhemoglobin (Hb) into oxyhemoglobin (HbO_2_) in tissues, which is related to angiogenesis, tumor cell proliferation, and hypoxia in tumor. Therefore, at present, DOS is considered another option in predicting early tumor response to neoadjuvant treatment [1, 7, 17, 19]. However, DOS test cycles in these studies are not representing all treatment cycles or the sample size is relatively small (patient number approximately ten).

The purpose of our study was to explore the change in blood-oxygen content using DOS to predict NAC response against breast cancer. We also evaluated the ability of these DOS parameters to analyze how early they can be detected or the accuracy they can achieve.

## Materials and methods

### Patients and NAC treatment

Written informed consent was obtained from all patients enrolled in this study and the study was approved by the institutional ethics committee of Affiliated Tumor Hospital of Guangxi Medical University. Twenty patients who had locally advanced breast cancer or planned to perform breast-conserving therapy were enrolled from June 2015 to August 2016. These patients received NAC 2–6 cycles before surgery. The chemotherapy regimen of seven patients in nonresponse group was changed to another effective regimen or they underwent surgery after two to six cycles. They underwent baseline clinical exam, mammogram, ultrasound, or MRI before treatment, and received at least two follow-up DOS scan. Oncologists and pathologists were blinded to DOS data. Cancer diagnosis was confirmed in all patients by core needle biopsy before NAC treatment. Ultrasound or MRI represented the baseline imaging for these patients. Ultrasound and clinical examination were performed at follow-up period. MRI was performed at presurgery (defined as the time within 1 week before surgery) for each patient. The tumor size and its change were recorded for each patient. Pathological characteristics were recorded after mastectomy, such as tumor size, histologic subtype, grade, and tumor response.

Chemotherapy was performed according to NCCN guideline(version 2.2016). Chemotherapy treatment regimen included AC (doxorubicin/cyclophosphamide) followed by docetaxel, TAC (docetaxel/doxorubicin/cyclophosphamide), FEC (fluorouracil/epirubicin/cyclophosphamide), FAC (fluorouracil/doxorubicin/cyclophosphamide), XEC (capecitabine/epirubicin/cyclophosphamide). Two patients with HER-2-positive tumor received trastuzumab with taxane. Doses and treatment regimen were adjusted according to toxicity and response.

### DOS system instrument

One type of DOS system was used, named E-sea (Multimode) Mammary Gland Blood-oxygen Functional Imaging System (ES-DM-X) (Wuhan E-sea Digital Technology Co., Ltd, Wuhan, China). ES-DM-X consists of blood and oxygen parameter detection and imaging in the diseased region of the mammary gland, and it displays abnormal blood vessels inside and outside diseased region. Within the blood-oxygen detection program, ES-DM-X can analyze blood-oxygen content in the tumor region and obtain related blood-oxygen images showed in the blood and oxygen content figure after the analysis.

The handheld probe was placed under the breast while the patient was sitting in a chair. Near-infrared light transmitted the skin and the parenchyma of the breast from the light source, it was collected by the optical imaging device and converted into electric signal. The tumor location and size in the volume of interest for blood-oxygen content images were similar with that in clinical exam and other imaging. DOS images of the breast lesion were obtained and blood-oxygen parameters in the breast lesion volume of interest could be directly obtained with ES-DM-X softscan platform.

### NAC response assessment

The first DOS scans were performed before the start of NAC and considered as baseline data (defined as pretreatment), and subsequent scans were performed at each circle after the start of NAC treatment or according to the actual follow-up situation of the patient. The follow-up DOS examination was defined as the measurements taken within 1 week before next treatment. The final scan was performed within 1 week before surgery (defined as presurgery).Two experienced radiographer (Yinghua Yu and Xiao Zhu) independently performed the DOS [(ES-DM-X)].

Tumor size and pathological examination analysis were included in our NAC response assessment. Tumor size was measured by clinical exam, ultrasound, mammogram, or MRI. According to the World Health Organization (WHO) criteria, tumor responses assessed by tumor size were as follows [10]: complete response (CR) meant that the tumor lesion disappeared completely and lasted for more than 4 weeks. If the tumor size decreased more than 50% and was stable for 4 weeks, it could be included in partial response (PR). If the tumor lesion decreased less than 50% or increased less than 25%, it was defined as stable disease (SD). Tumor responses assessed by pathological examination after NAC were classified in three categories [17]: pathological complete response, pathological good response, or pathological minimal response. (1) Pathological complete response was defined as no residual malignancy or in situ tumor presence [9]. (2) Pathological good response was defined as a tumor size reduction over 50% with the invasive cells less than 10%. (3) Pathological minimal response was defined as minimal invasive tumor reduction. Patients who obtained CR or PR in radiological definitions and complete or good pathological response as demonstrated by pathological assessment were included in the response group. The remaining patients were included in the nonresponse group.

### Statistical analysis

Statistical analysis was calculated using SPSS 13.0 software (Chicago, IL, USA). Blood and oxygen content and tumor size were not normally distributed (Kolmogorov–Smirnov test, *P* < 0.05). Therefore, changes in blood and oxygen content value were compared using two-way non-parametric Mann–Whitney test between two groups such as pretreatment and presurgery, response and nonresponse groups. *P* < 0.05 was considered statistically significant. Diagnostic parameters such as sensitivity, specificity, and the area under the ROC curve (AUC) were applied to assess the diagnostic performance in differentiating tumor response from nonresponse.

## Results

### Patients’ characteristics

Twenty female patients, 31–62 years old (median age 45.9 years) were recruited in this study. According to our NAC treatment response criterion, 13 patients were classified into response and 7 patients into nonresponse group. Patient’s age, tumor size and stage, pathology, primary neoadjuvant regimen, number of DOS follow-up scan, blood and oxygen content parameter were measured and listed in Table 1.

**Table 1:** Clinical characteristics of 20 patients

Group and patient no.	Age (years)	Cancer type	Initial tumor size (cm)	Tumor stage	Biomarker status (positive/negative/NA)			Follow-up cycle (*n*)	Primary neoadjuvant	Pretreatment		Presurgery	
					Estrogen receptor	Progesterone receptor	HER-2			BCV	OCV	BCV	OCV
Responder													
1	35	IDC	7.8	IIIA	Negative	Negative	Negative	4	TAC	−0.54	0.91	0.09	1.28
2	51	IDC	4.5	IIB	Positive	Positive	Negative	4	EC → T	−1.59	0.33	−0.84	0.46
3	35	IDC	4.4	IIA	Negative	Negative	Positive	3	AC → TH	−1.24	0.43	−0.11	0.92
4	45	IDC	5.4	IIIA	Negative	Positive	Negative	4	FEC	−0.67	0.58	−0.25	0.86
5	42	IDC	6.5	IIIA	Positive	Negative	Negative	5	AC → T	−1.58	0.41	1.00	0.55
6	31	IDC	9.0	IIIB	Negative	Negative	Negative	6	AC → T	−0.93	0.43	−0.07	0.98
7	47	IDC	5.8	IIB	Positive	Negative	Positive	6	AC → TH	−1.48	0.31	0.55	1.49
8	36	IDC	7.5	IIIA	Negative	Negative	Negative	6	TAC	−1.31	0.39	−0.24	0.81
9	41	IDC	4.6	IIB	Negative	Positive	Positive	6	EC → T	−0.72	0.57	−0.07	0.88
10	45	IDC	6.5	IIIA	Negative	Positive	Negative	4	TAC	−1.30	0.37	−0.95	0.54
11	53	IDC	6.9	IIB	Positive	Negative	NA	2	AC → T	−1.40	0.32	−1.10	0.35
12	47	IDC	5.4	IIIA	Negative	Positive	Negative	3	AC → T	−0.49	0.69	−0.01	0.96
13	62	IDC	5.8	IIIA	Positive	Negative	Negative	6	TAC	−0.50	0.55	0.10	1.30
Non-responder													
14	42	IDC	6.0	IIIA	Positive	Positive	Negative	6	AC → T	−1.77	0.37	−0.62	0.63
15	41	IDC	4.2	IIB	Negative	Negative	Positive	2	EC → T	−0.89	0.47	−0.72	0.55
16	45	IDC	6.0	IIIA	Negative	Negative	Negative	4	AC → T	−0.98	0.38	−0.77	0.54
17	49	IDC	3.9	IIB	Negative	Positive	Negative	3	XEC	−0.41	0.65	−0.21	0.72
18	64	IDC	3.5	IIIA	Positive	Negative	Negative	2	ACT	−1.49	0.16	−1.58	0.22
19	50	ILC	8.0	IIIA	Negative	Negative	Negative	3	TAC	−0.27	0.95	−0.33	0.72
20	58	IDC	5.8	IIB	Positive	Positive	NA	3	FAC	−1.69	0.35	−1.52	0.47

*IDC* invasive ductal carcinoma, *ILC* invasive lobular carcinoma, *NA* not available, *n* number, *BCV* blood content value, *OCV* oxygen content value

### Blood and oxygen content in patients after NAC treatment

**Fig. 1:**
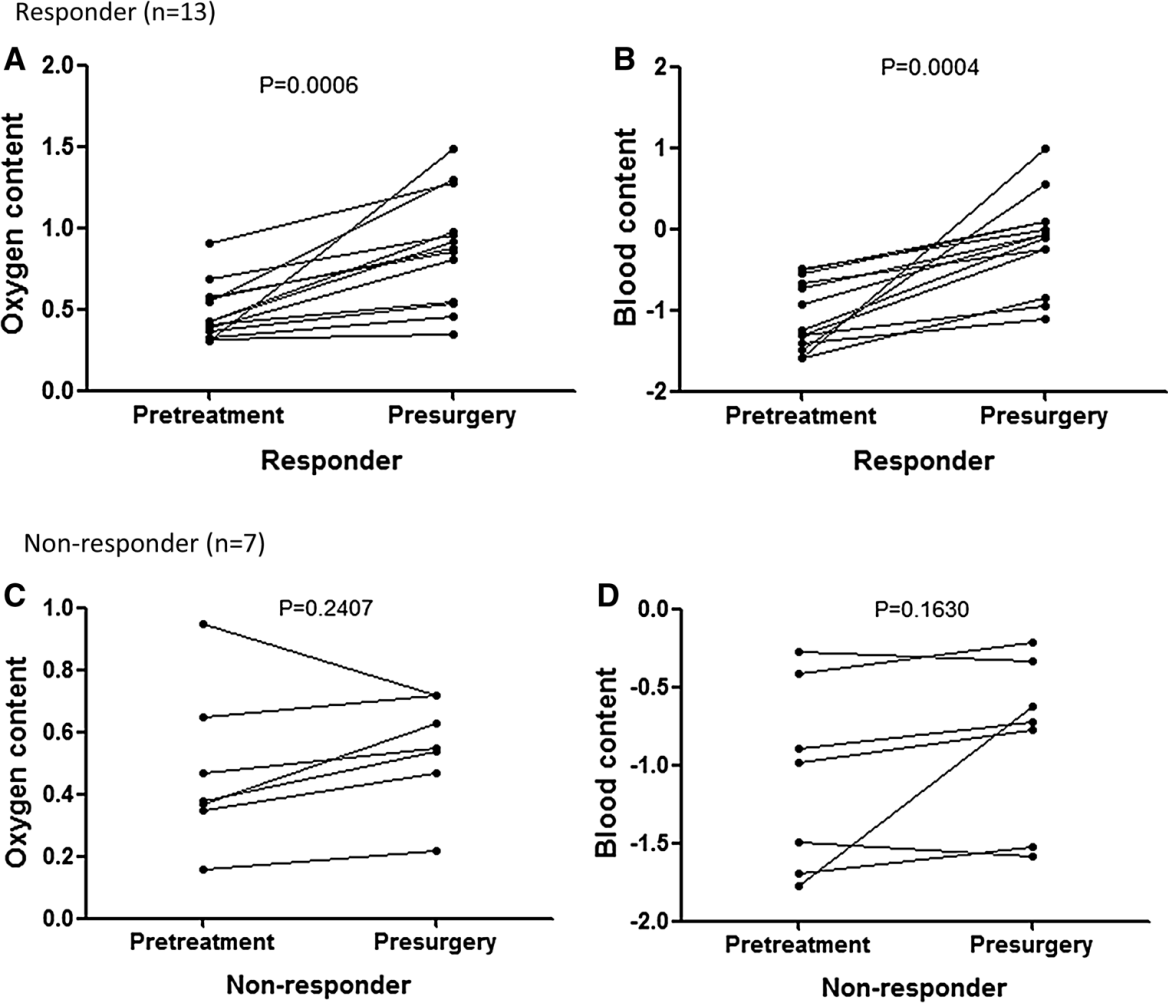
Plots of changes in tumor blood-oxygen content for individual patients, stratified in responder and non-responder by final pathological response. The plotted value is blood-oxygen content. The pretreatment and presurgery points correspond to the measurements taken within 1 week before chemotherapy treatment and within 1 week before surgery, respectively. The differences of the oxygen (**a**, **c**) and blood (**b**, **d**) content groups were expressed by negative and positive values in ES-DM-X system, respectively. The larger the positive value, the higher the oxygen value. On the contrast, the larger the negative value, the higher the blood value. The changes of responder are distinctive from non-responders in the neoadjuvant chemotherapy treatment

Figure 1 shows the changes referred to blood-oxygen content after NAC treatment, divided into response group and nonresponse group according to the pathological response classification. Each line represents one patient, where the two points correspond to DOS measurements taken within 1 week before chemotherapy treatment and within 1 week before surgery, respectively. The points are the value of a DOS parameter averaged over the tumor region. The differences in oxygen and blood content were expressed by negative and positive values in ES-DM-X system, respectively, thus different from other DOS system types. In the ES-DM-X system, the higher the oxygen content value, the larger the positive value. On the contrary, the larger the negative value, the higher the blood content value. Figure 1 and Table 2 summarized NAC effects on blood and oxygen content parameters. The blood content value at pretreatment (−1.06 ± 0.43) was significantly different than presurgery (−0.15 ± 0.58) in the response group (*P* = 0.0004). On the contrary, blood content value between pretreatment (−1.07 ± 0.60) and presurgery (−0.82 ± 0.54) was not statistically significant in the nonresponse group (*P* = 0.1630). The oxygen content value at pretreatment (0.48 ± 0.17) was significantly different than presurgery (0.88 ± 0.34) in the response group (*P* = 0.0006). On the contrary, the oxygen content value between pretreatment (0.48 ± 0.26) and presurgery groups (0.55 ± 0.17) was not statistically significant in nonresponse group (*P* = 0.2407).

**Table 2:** Differences of blood and oxygen content between responder and non-responder

Parameter	Responder (Mean ± SD)		*P*	Non-responder (Mean ± SD)		*P*
	Pretreatment	Presurgery		Pretreatment	Presurgery	
Blood content	−1.06 ± 0.43	−0.15 ± 0.58	0.0004	−1.07 ± 0.60	−0.82 ± 0.54	0.1630
Oxygen content	0.48 ± 0.17	0.88 ± 0.34	0.0006	0.48 ± 0.26	0.55 ± 0.17	0.2407

Mann–Whitney test

### Percentage changes in blood content, oxygen content, and lesion size in response and nonresponse group

**Fig. 2:**
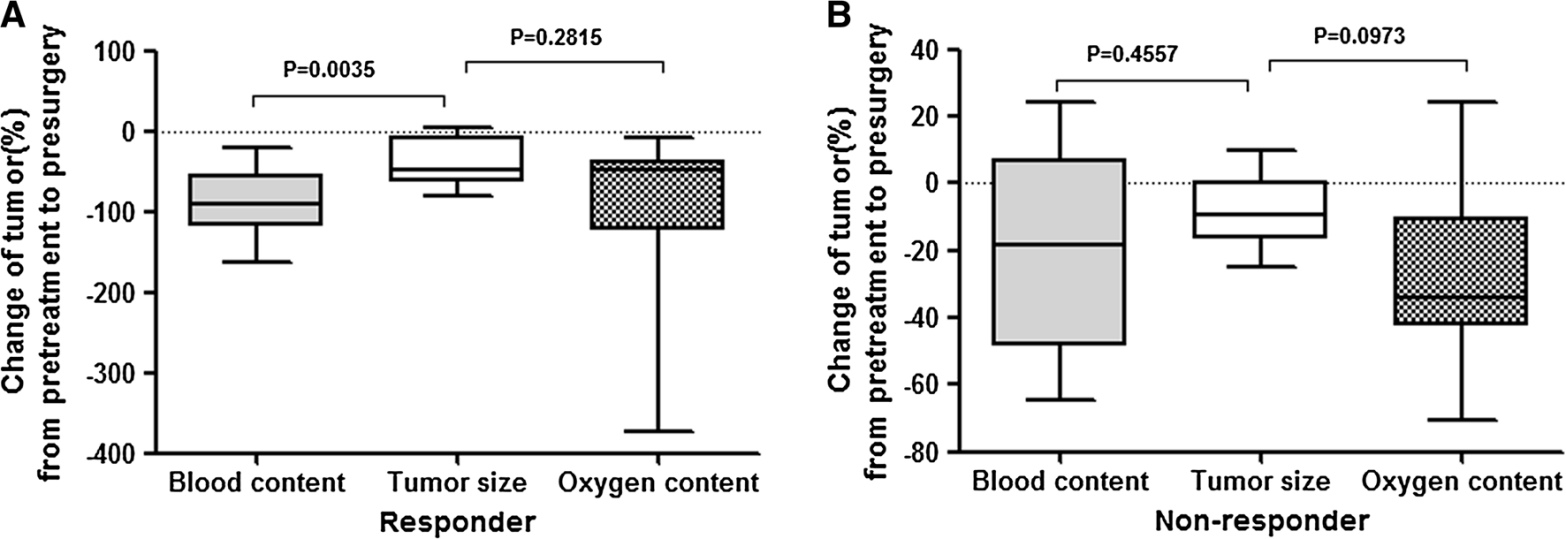
*Boxplot* shows comparison of percentage changes in tumor size, blood content, and oxygen content value during NAC treatment (from pretreatment to presurgery) in response and nonresponse groups. Tumor size was regarded as a control standard. **a** In response group, the percentage change of blood content value decreases significantly than tumor size. While the percentage change of oxygen content is not statistically different from tumor size. **b** In nonresponse group, there were not significantly larger reductions in blood and oxygen content value compared with tumor size

Figure 2 shows the percentage changes in blood content, oxygen content, and tumor size during NAC treatment (from pretreatment to presurgery) in response and nonresponse groups. Percentage change in blood content (median 91.19%) was significantly larger than tumor size (median 48.89%) in response group (*P* = 0.0035). Although the percentage change in oxygen content (median 47.11%) was not statistically different from tumor size (*P* = 0.2815), oxygen content changed more than tumor size. Not significantly larger percentage change in blood and oxygen content percentage were observed in nonresponse group compared with tumor size (*P* > 0.05).

**Fig. 3:**
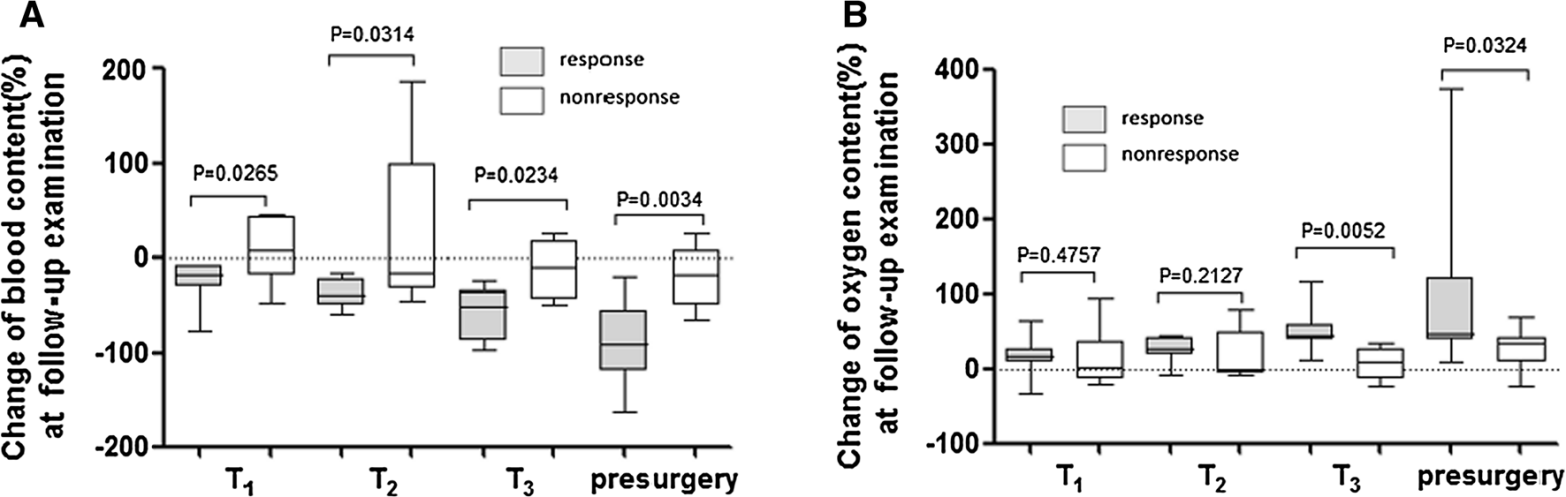
*Boxplot* shows percentage change of blood and oxygen content changes comparing response with nonresponse group at first, second, third, and presurgery (defined as the examination before surgery) follow-up. **a** Percentage change of blood content at follow-up examination; **b** change of oxygen content at follow-up examination. The percentage change of blood content value was all significantly larger in response group than in nonresponse group at these four follow-up examination. The change of oxygen content value was significantly larger in response group than in nonresponse group at third and presurgery follow-up examination. *T*_*1*_ first follow-up, *T*_*2*_ second follow-up, *T*_*3*_ third follow-up, *presurgery* the last follow-up before surgery

As illustrated in Tables 3 and 4, baseline (defined as pretreatment data) tumor size, blood content, and oxygen content value were not significantly different between response and nonresponse group. Blood content percentage change was significantly larger in the response group compared with nonresponse group at first (19.1 versus 6.6%, *P* = 0.0265), second (38.91 versus 15.87%, *P* = 0.0314), third (51.14 versus 10.23%, *P* = 0.0234), and presurgery (91.19 versus 18.47%, *P* = 0.0034) follow-up, while the percentage change in oxygen content was not significantly different between response and nonresponse group (*P* > 0.05) until the third (45.1 versus 10.40%, *P* = 0.0052) and presurgery (47.11 versus 33.70%, *P* = 0.0324) follow-up examination (Fig. 3; Table 3). Tumor size percentage change in response group was significantly larger than in nonresponse group (*P* = 0.0315) at presurgery follow-up examination (Table 4).

**Table 3:** Compared the percentage change in blood and oxygen content at follow-up examination

Change at Follow-up examination	Blood content			Oxygen content		
	Responder	Non-responder	*P* value	Responder	Non-responder	*P* value
Pretreatment (baseline)	−1.24 (−1.59, −0.49)	−0.98 (−1.77, −0.27)	0.8741	0.43 (0.31, 0.91)	0.38 (0.16, 0.95)	0.8428
First follow-up (%)	−19.10 (−76.12, −8.04)	6.66 (−46.03, 44.67)	0.0265	17.23 (−34.25, 63.65)	1.180 (−21.43, 94.55)	0.4757
Second follow-up (%)	−38.91 (−57.67, −16.86)	−15.87 (−45.24, 187.4)	0.0314	25.69 (−9.430, 44.19)	−0.5100 (−9.160, 79.28)	0.2127
Third follow-up (%)	−51.14 (−97.28, −24.29)	−10.23 (−48.21, 24.34)	0.0234	45.10 (11.34, 115.6)	10.40 (−24.03, 33.70)	0.0052
Presurgery (%)	−91.19 (−163.2, −21.47)	−18.47 (−64.83, 24.34)	0.0034	47.11 (8.940, 373.8)	33.70 (−24.03, 70.34)	0.0324

Numbers in parentheses are the median, followed by the range of minimum to maximum. Significance (*P* < 0.05) between response and nonresponse group tested using two-way Mann–Whitney nonparametric test. The follow-up examination was defined as the measurements taken within 1 week before next treatment

**Table 4:** Percentage changes in tumor size between response and nonresponse group

Tumor size	Responder	Non-responder	*P* value
Pretreatment [baseline (cm)]	4.3 (3.0, 15.0)	3.6 (2.0, 8.0)	0.1768
Change at presurgery (%)	−48.89 (−81.4, 4.76)	−9.68 (−25.00, 10.00)	0.0315

Numbers in parentheses are the median, followed by the range of minimum to maximum. Significance (*P* < 0.05) between response and nonresponse group tested using two-way Mann–Whitney nonparametric test

### Prediction NAC treatment response

Table 5 shows the diagnostic predicted performance in blood content, oxygen content, and tumor size parameters between response group and nonresponse group. The optimal threshold of blood content, oxygen content, and tumor size value was determined to distinguish response from nonresponse. The sensitivity and specificity were calculated according to the optimal threshold that can predict the final tumor response.

**Table 5:** Diagnostic performance of blood content, oxygen content, and tumor size for the prediction of treatment response

Percentage change (from pretreatment to presurgery)	Sensitivity	Specificity	Cutoff (%)	AUC (95% CI)
Blood content percentage change (%)	0.769	0.857	0.56	0.912 (0.786, 1.000)
Oxygen content percentage change (%)	0.769	0.714	0.39	0.797 (0.591, 1.000)
Tumor size percentage change (%)	0.692	0.857	0.21	0.802 (0.607, 0.997)

Blood content percentage change achieved a sensitivity of 76.9% and a specificity of 85.7% (AUC 0.912 (95% CI 0.786–1.000); cutoff, 55.5%). Oxygen content percentage change yielded a sensitivity of 76.9% and a specificity of 71.4% (AUC 0.79.7 (95% CI 0.591–1.000); cutoff, 39%). Tumor size percentage change achieved a 69.2% sensitivity and 85.7% specificity (AUC 0.802 (95% CI 0.607–0.997); cutoff, 0.210%).

### Case study

**Fig. 4:**
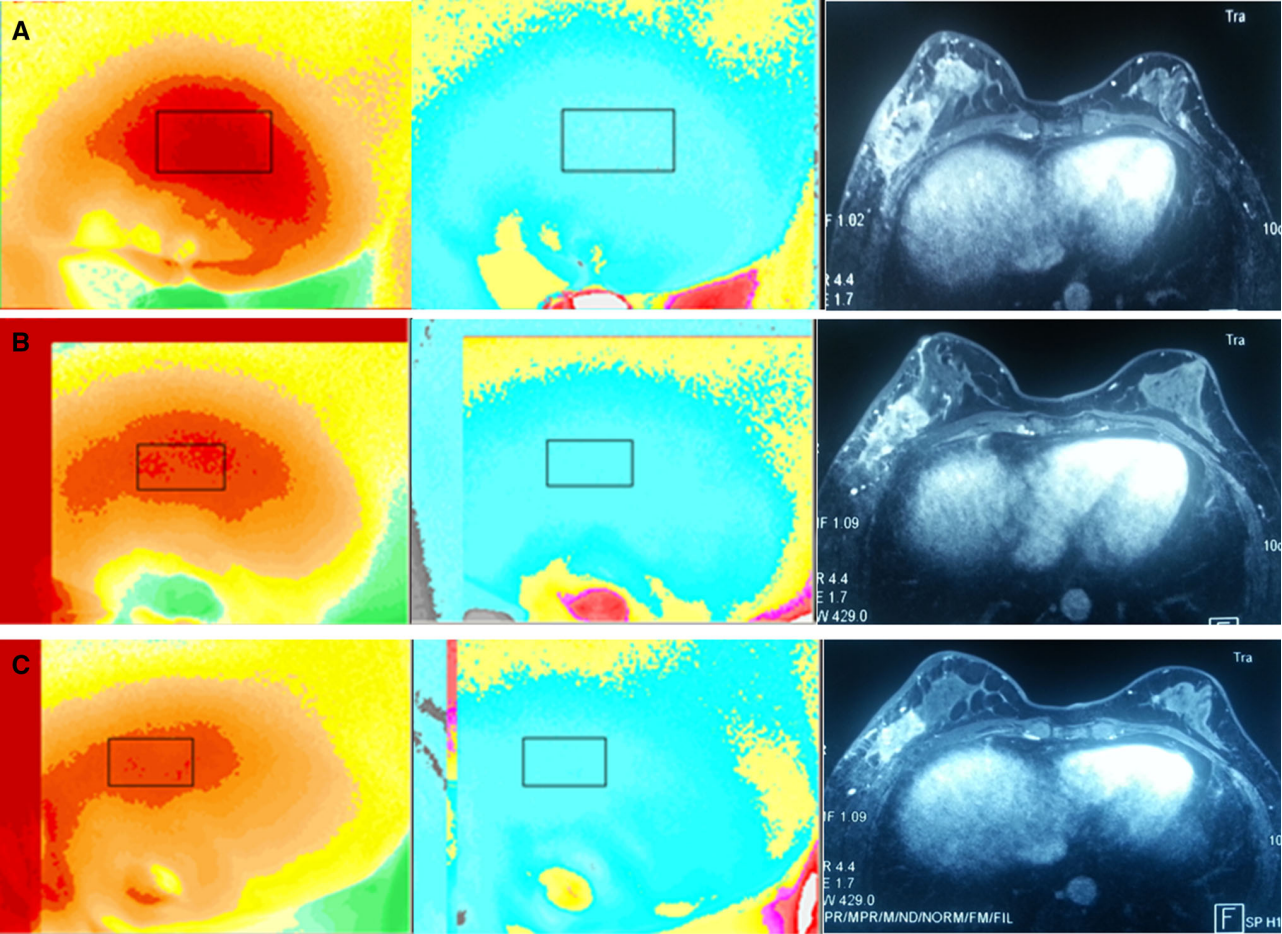

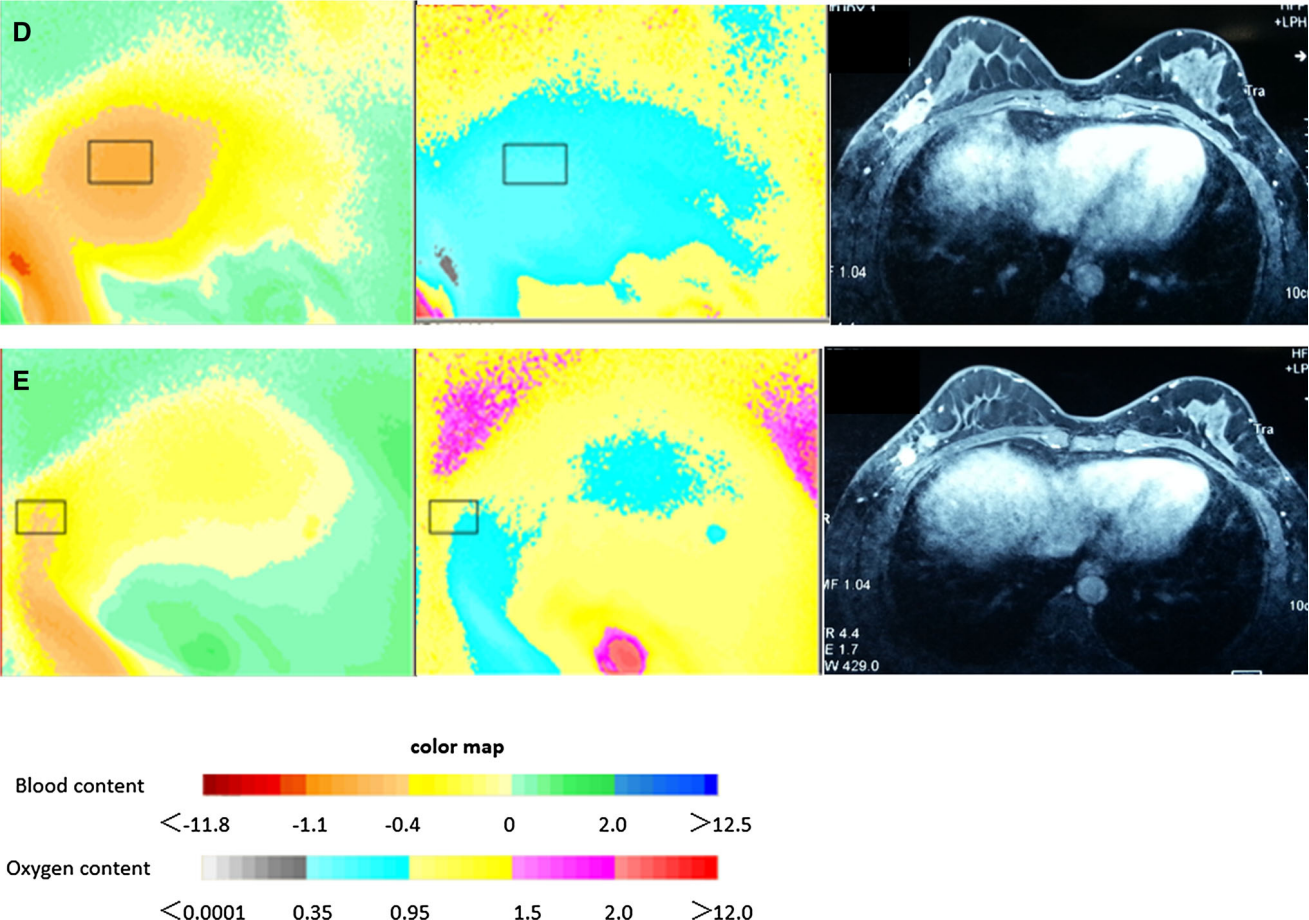
The corresponding blood-oxygen images and MRI of a typical breast cancer responder who completed five NAC treatment cycle before surgery. The figures from A to E present the change of the malignant tumor in blood-oxygen images and MRI. Left, blood value image; median, oxygen value image; right, MRI image. The colormap shows the parameter of blood-oxygen content of tumor lesion. In blood value image, *Red area* presents high blood content; in oxygen value image, *blue area* means low oxygen content. The image of malignant tumor always presents high blood and low oxygen. It was presented that the blood content decreased and the oxygen content increased, which was a typical responder’s change. As a control, the tumor size in MRI became more and more small

Figure 4 shows typical blood-oxygen images and MRI of a 41-year-old responder. The patient completed five NAC treatment cycles before surgery. Malignant tumor DOS image showed high blood content and low oxygen content (Fig. 4a left and median image). The blood content decreased and the oxygen content increased following each treatment cycle (Fig. 4b–e left and median image), which was a typical responder’s change trend according to the physiology of malignant tumor change, corresponding to a smaller and smaller tumor size as shown by MRI (Fig. 4 right image). The change in blood and oxygen content was synchronous with that of tumor size.

## Discussion

NAC is an effective treatment against LABC before mastectomy or breast-conserving therapy. However, it is of utmost importance an early response evaluation for predicting whether NAC should be continued before surgical intervention.

It had been accepted that the change in tumor size measured on ultrasound, X-ray or MRI, could predict pathological responses after NAC [13]. Change in tumor size takes time (for example at least after two to three treatment cycles) [1]. Therefore, some functional methods [14], such as contrast-enhanced ultrasound [11], MRI [16] and fluorodeoxyglucose (FDG PET) [6], have been researched to assess NAC treatment response. However, contrast-enhanced ultrasound was invasive and still pending for approval of microbubble contrast agents for this indication. MRI was invasive to inject contrast agent and relatively costly. Furthermore, no functional standard criteria on MRI have yet emerged to evaluate NAC treatment response. The cost of FDG PET limited its widespread application.

Measuring oxyhemoglobin content can reveal the content of oxygen in blood, thus revealing body tissue metabolism. Deoxyhemoglobin absorbance is different from that of oxyhemoglobin [22] at 600–800 nm wavelength. The red light absorbed by hemoglobin in angiogenic regions, can reflect the difference between non-blood vessel areas and blood vessel areas. Hence, tumor response could be evaluated if the changes due to re-distribution of blood volume and oxygenation level are detected. DOS could assess tumor response by analyzing oxyhemoglobin (HbO2) and deoxyhemoglobin (Hb) change in tissue [1, 17].

Our study adopted a new type of DOS system named ES-DM-X system (Wuhan E-sea Digital Technology Co., Ltd, Wuhan, China). The change in blood-oxygen content could be directly perceived from the screen image of ES-DM-X system. According to the blood-oxygen content image and the relative blood-oxygen content parameter after each NAC cycle, the doctor is able to estimate as early as possible if NAC regimen fits for the patient. This kind of examination is safe and noninvasive because of the absence of radiation damage and without contrast medium injection. Furthermore, it is relatively not expensive and requests less time to perform image examination than other systems. Thus, measurements can be repeated as many times as needed with no harm to patients, resulting in a more easily accepted scanning method by patients during the treatment response monitor period. Although the blood-oxygen content parameter measurement in ES-DM-X system is different from that of other DOS system, ES-DM-X system detection principle was similar with other systems and blood-oxygen image was more clearly visualized than the other DOS systems.

Our study used clinical or pathologic response (response versus nonresponse) as the endpoint, which was also adopted by previous studies [1, 17]. The response and nonresponse group showed different response patterns in blood-oxygen content change. Comparing each cycle, the percentage change in blood content could distinguish responder from non-responder as early as after the first treatment cycle in response group (Fig. 3; Table 3). In other words, blood content could be a sensitive method to detect treatment response. However, oxygen percentage content value was not significantly modified until after three examining cycles, suggesting that change in oxygen content was not as sensitive as blood content. When comparing the lesion size and blood-oxygen change in response group, blood content parameter changed more dramatically than tumor size and oxygen content in response group, revealing its good tumor response prediction with NAC treatment compared with the information given by tumor size or oxygen content. The blood content percentage change was the highest among oxygen content and tumor size at the scan before surgery in response group (Fig. 2a), meaning that change in blood content could be more easily monitored than other parameter in response group. There is a need to mention that our pretreatment blood content and oxygen content value were not significantly different between response and nonresponse group (Table 3), while a new publication demonstrated that the pretreatment DOS texture features can predict breast cancer response to NAC [19]. The reason may be that our study adopted a new type of DOS system named ES-DM-X system, and the blood-oxygen content parameter measurement in ES-DM-X system is different from that of other DOS system. The limited sample size is the limitation in both studies. Thus, a larger prospective study is needed to improve the present different results. After all, both studies confirms that DOS system could predict early therapeutic response.

We finally analyzed the diagnostic performance of blood content, oxygen content, and tumor size for predicting tumor response with NAC treatment (Table 5). The change in blood content percentage received an AUC of 0.921, which was the highest among other parameters such as tumor size and oxygen content, indicating that the blood content percentage change may be the optimal predictor of tumor response with NAC treatment. The AUC of oxygen content percentage change was similar to that of tumor size. Thus, change in oxygen content percentage could also be used as an alternative method to analyze tumor response. Therefore, DOS image (ES-DM-X) including blood-oxygen content could provide an alternative approach to assess breast tumor response, which represented a supplementary measurement besides tumor size. According to our analysis, blood content was more precise and earlier than oxygen content to predict tumor response. Compared to other imaging modalities, AUC of blood and oxygen content percentage change in the response and nonresponse group were similar with those obtained by quantitative ultrasound (QUS) (AUC of 81%) [18], or even higher than PET/CT (AUC of 63.6%) or MRI (AUC of 75.7%) [3]. Although we could not demonstrate that blood and oxygen content by DOS (ES-DM-X) was more precise than QUS, PET/CT and MRI, it could be speculated that they all have the same power to predict tumor response after NAC treatment. A typical blood-oxygen images and MRI of a 41-year-old responder (Fig. 4) directly show that the change in blood and oxygen content was synchronous with that of tumor size in MRI.

However, our study still had some limitations. It included patients with many different clinical characteristics and different NAC treatment protocols, although this aspect is common in oncology research. In addition, this work lacks standard criteria to perform the blood-oxygen content test or analyze the result, for example to evaluate how long or how often the blood-oxygen content should be tested, and which blood-oxygen content is the best threshold to evaluate the positive or negative result. Thus, a larger prospective study is needed to improve the present result.

In conclusion, our data indicated that blood content could detect the breast tumor response as early as after the first treatment, and both blood and oxygen content could be used to discriminate between responder and non-responder after NAC treatment. Although blood content was better than oxygen content to predict tumor response, they all could be supplementary choices besides measuring tumor size.

## Abbreviations

DOS: Near-infrared diffuse optical spectroscopy
NAC: Neoadjuvant chemotherapy
pCR: Pathologic complete response
AC: Doxorubicin and cyclophosphamide
HER-2: Human epidermal growth factor receptor 2
AUC: Area under the ROC curve
ROC: Receiver operating characteristic
LABC: Locally advanced breast cancer
MRI: Magnetic resonance imaging
PET/CT: Positron emission tomography/computed tomography
NAC: Neoadjuvant chemotherapy
CR: Complete response
PR: Partial response
SD: Stable disease
Hb: Deoxyhemoglobin
HbO2: Oxyhemoglobin
QUS: Quantitative ultrasound
